# 

**Published:** 1857-07

**Authors:** 


### TO LOCAL OBSERVERS OF DISEASE.

Sets of Ozone papers and scales have this quarter been forwarded to various local observers of epidemic diseases. The instructions to those gentlemen, who have kindly undertaken to labour in this department, are few and simple.

1. Every morning at 9 o'clock, put an Ozone test paper in a place freely exposed to the air, but protected from light and atmospheric currents. At 9 o'clock the following morning, remove the paper, and put another in its place, which, in its turn, remove at 9 o'clock the next morning, and so on. At the time of removal, breathe upon the slip of paper, compare it with the scale, and register the number with which it corresponds.

2. At the end of each week, ending on Friday, uniform with the weeks marked out in the report sheet, let the result of each day's observation be compared and the mean taken. Then simply register the mean figure in the report sheet, in the division for the proper week, to the right of the + sign.

To secure uniformity of results, observers will please commence observations on Friday the 10th of July ensuing.

Any additional notes, in relation to the presence or absence of ozone in connection with winds blowing from various quarters, will be of great value.

---

### OBSERVATIONS ON AGUE.

The prevalence of ague in various districts, as described in last Report, is a fact of peculiar interest. The editor would be obliged, if Dr. Brown of Chatham, Dr. Barker of Bedford, Mr. Rigden of Canterbury, Mr. Hole of Wisbech, Mr. Swann of Barrowdon, and other observers, would in the next Review report specially on the probable causes and nature of this disease. The editor respectfully suggests for solution the following queries :—

1. Do any general facts, connected with the cases, tend definitely to connect them with impure drinking water as their cause?

2. If such be the fact, and if, in any given instance, a series of cases can be traced back as originating from bad water; in what does the impurity of the water consist? What is its source? What are its usual qualities?

3. Granting that no connection between ague and water supply can be traced, is there any positive evidence of an origin from an atmospheric cause; from malaria, or miasm?

4. What positive facts have cases observed taught, as to the relative merits of quinine and arsenic as curative agents?

5. Will quinine positively cure ague, irrespective of all hygienic rules and precautions? Are there any known hygienic rules, which strictly carried out, will alone remove ague, or assist materially in its removal?

6. Has any observer ever met with a case, or cases, where recovery has occurred spontaneously, *i. e.*, without the aid of medicines. If so, were any peculiar circumstances noted which would account for the cure?

7. Does the occurrence of one attack of the disease ever seem to defend the patient, in the slightest degree, from a second attack?

8. Does observation of the disease completely exclude all suspicion that the disease is communicable from person to person?

9. Does observation confirm Dr. Ranke's late interesting statements; viz., that in ague there is an increase of uric acid in the urine; and that under quinine the quantity of uric acid in the urine is diminished?

---

### NOTES AND CORRESPONDENCE.

The Editor is anxious to give free scope to the expression of opinion, but does not hold himself responsible for the opinions of those of his contributors who attach their names to the *papers furnished* by them.

The Tucker Fund. We step willingly for once out of our usual course, to call the attention of readers to the case of the benevolent Founder of the Epidemiological Society, Mr. J. H. Tucker. It is possible that the public sympathy was never solicited on more telling evidence. If the exertions of this estimable and self-sacrificing man were known as well to all, as they are to us who knew him in his labours, this appeal would indeed be a success. Observing that all great epidemics are under the influence of fixed, but unknown laws, it occurred to Mr. Tucker, that if many minds were directed to the investigation of these laws, some new light might break forth here or there, and that in the development of even a single and simple fact, some great modern pestilences might be brought to an end. Thus instinctively instructed, he suggested the idea of a special society for investigating epidemics. He enlisted the aid of the right men, and achieved even a greater success than he had foreseen.

What he had thus happily commenced he too earnestly, for his own sake, pursued. The Society was his everything; he worked for it incessantly at a period of life when the mind wants rest.

He paid for it beyond his means. The labour at last bent down the man; and now, worn out in body and mind, our poor friend, without any prospect of recovery, is left entirely dependent, for the means of existence, on the benevolence of his countrymen.

But we should omit much real claim, did we not say that the man himself was the epitome of genuine worth, liberality, and benevolence. Did a brother want assistance, his sympathies were awakened, and his hand was doing. Did the poorest ask him for his professional aid, he was at once by the bedside—his best knowledge at the poorest one's command. Had this man been a sham, and had he done nothing but pretend, the State perchance might have been appealed to with success, and have recognised his claims at the worth of at least a hundred a year, during the rest of his days. As it is—as the man was good, generous and pure; as he has done an important service to his country, which will grow in time till men are amazed at its results—this hope of relief is of course entirely cut off. But, surely, an appeal will not be in vain to those who believe that there are as yet extant specimens of men real and single-minded, and that these should not always die without recognition.

Any Subscriptions for the Tucker Fund that may be sent to the Editor of the Sanitary Review, will be forwarded by him to the Committee who have the management of the Fund.

---

### *Dr. Hawksley on Mr. Rogers's Drainage Plan.*

From a pamphlet of Mr. Jasper W. Rogers, C.E., sent to me about a month since, entitled "Facts and Fallacies of the Sewerage System of London," I learn that he, Mr. Rogers, exhibited a plan very similar to mine (as recorded in your last number) to the Commissioners of Sewers in 1849, and that he published it, *in extenso,* in the *Morning Herald* of August 23rd, 1849. I need scarcely say, that I and all the friends with whom I had conversed on the subject, were ignorant of these facts up to the time Mr. Rogers's pamphlet was sent to me, as described.

His proposal is full of originality, and especially commendable for its completeness; I am astonished that its publication excited so little inquiry, and that its important pretensions were not subjected by our rulers to the test of experiment.

There are some other differences between Mr. Rogers's plan and mine; instead of a filtering 'receptacle,' he adopts a filtering 'tank' which fits into a receptacle, and out of which it (the tank) may be lifted and conveyed away when filled with the solid residue of its filtering action. Again, instead of a screen of gravel to filter through, he employs a screen of granulated charcoal. There are also many other minor differences, but the principle in both is the same, and I award to him the priority.—Thomas Hawksley.

---

### BOOKS RECEIVED.

ACTON (William). The Functions and Disorders of the Reproductive Organs. London: Churchill, 1857.

BALLARD (Edward, M.D.) Report on the Sanitary Condition of Islington in 1856. London: 1857.

BARCLAY (A. W., M.D.) Report on Sanitary Condition of St. Luke's, Chelsea. Chelsea: J. MacMichael, 1857.

BAKEWELL (R. H., M.D.) Practical Hints on the Management of the Sick Room. London: Snow, 1857.

BLYTH (Lindsay). Minute of Information on Disinfection and Deodorization. Published for the Board of Health. London: 1857.

COCKLE (John, M.D.) On the Second Sound and Murmur of the Heart and Great Bloodvessels. London: Richards, 1857.

DICKENS (Alfred L., C.E.) Reports on a Preliminary Inquiry into Sanitary Condition of Droitwich, and of Seaford. Further Inquiry at Windhill and Leamington Priors. Board of Health Reports. London: 1857.

DOBELL (H., M.D.) On the Class of Medical Literature most needed at the Present Day. London: 1857.

FELL (J. W., M.D.) A Treatise on Cancer and its Treatment. London: Churchill, 1857.

GAMGEE (Joseph S.) The Cattle Plague, and Diseased Meat. Two Letters to the Right Hon. Sir George Grey, Secretary of State for the Home Department. London: Richards, 1857.

GARRETT (C. B., M.D.) On the Causes, Symptoms, and Treatment of Irritative Congestion of the Windpipe. London: Churchill, 1857.

GLAISHER (James, F.R.S.) On the Meteorology of England and of Scotland during the Quarter ending March 31st, 1857. London: 1857.

GREENHOW (E. H., M.D.) Report on Murrain in Horned Cattle, and the Public Sale of Diseased Animals. Blue Book. London: 1857.

GRIFFIN (Richard) Statement of the Grievances of the Poor Law Medical Officers. Weymouth: Archer, 1857.

HALL (C. Radclyffe, M.D.) Torquay in its Medical Aspect as a Resort for Pulmonary Invalids. London: Churchill, 1857.

HARE (S.) Cases of Spinal Deformity, with Observations. London: Churchill, 1857.

HASSALL (A. H., M.D., F.L.S.) Report on the Microscopical Examination of the Metropolitan Water Supply. Government Report. London: 1857.

HILLIER (Thomas, M.D.) First Annual Report of the Medical Officer of Health of St. Pancras, Middlesex. London: 1857.

HOOD (W. C., M.D.) Statistics of Insanity; being a Decennial Report of Bethlem Hospital, from 1846 to 1855, inclusive. London: Batten, 1857.

ILIFF (W. T., M.D.) Report on the Sanitary Condition of St. Mary Newington, Surrey. London: 1857.

LETHEBY (H., M.B.) Report on the Sanitary Condition of the City of London for the Quarter ending March 20th, 1857. London: 1857.

MILLAR (John). A Refutation of the Fourth Annual Report of the Visitors of the Bucks County Lunatic Asylum, so far as regards the Matter of Change of Superintendent. London: Renshaw, 1857.

NORTHCOTE (A. B., F.C.S.) On the Constitution of Allophane. London: 1857.

REPORT on the Nuisances arising from Gas Works. By the Trades Committee of the Metropolitan Association of Medical Officers of Health. London: Spottiswoode, 1857.

ROBERTON (John). On Certain Legalized Forms of Temptation as Causes of Crime. [Reprint from Manchester Statistical Society Transactions.] Manchester: 1857.

SAYER (H., M.D.) On Metropolitan and Town Sewage, their Nature, Value and Disposal. London: Calder, 1857.

URLING (G. F.) Vocal Gymnastics, or a Guide for Stammering. London: Churchill, 1857.

WEBSTER (John, M.D., F.R.S.) Notes on Belgian Lunatic Asylums, including the Insane Colony of Gheel. [Reprint from Psychological Journal.] London: 1857.

WEEKLY RETURNS on the Rise and Decline of Disease in the Metropolis. Published by the Board of Health. London, 1857.

### IN EXCHANGE.

Literary Gazette.
Psychological Journal.
British Medical Journal.
Ranking and Radcliffe's Abstract of the Medical Sciences.
Chemist.
Veterinarian.
Asylum Journal of Mental Science.
Quarterly Journal of the Chemical Society.
Scottish Review.
Edinburgh Medical Journal.
Glasgow Medical Journal.
Dublin Hospital Gazette.
American Journal of the Medical Sciences
New York Journal of Medicine.
Montreal Medical Chronicle.
Charleston Medical Journal.
Gazette Médicale de Paris.
Aerzliches Intelligenz-Blatt.

### To New Subscribers to the Sanitary Review.

THE Publisher begs to intimate, that new Subscribers wishing to have the series of the Journal complete from the first number, can obtain sets of Vols. I and II, at Subscribers' prices, 10s. each, post free, by application to him, enclosing Post-office Order, payable at the Charing Cross Branch to THOMAS RICHARDS.

THE SANITARY REVIEW AND JOURNAL OF PUBLIC HEALTH is sold by all Booksellers, and is equally suited for the general and professional reader, for the scientific society, and for the private library.

London: T. RICHARDS, 37, Great Queen Street.

